# Nerve Growth Factor (NGF) modulates in vitro induced myofibroblasts by highlighting a differential protein signature

**DOI:** 10.1038/s41598-021-81040-x

**Published:** 2021-01-18

**Authors:** Graziana Esposito, Bijorn Omar Balzamino, Egidio Stigliano, Filippo Biamonte, Andrea Urbani, Alessandra Micera

**Affiliations:** 1 Research and Development Laboratory for Biochemical, Molecular and Cellular Applications in Ophthalmological Sciences, IRCCS-Fondazione Bietti, Rome, Italy; 2 Fondazione Policlinico Universitario A. Gemelli, IRCCS, 00168 Rome, Italy; 3 Istituto di Biochimica e Biochimica Clinica e Istituto di Anatomia Patologica, Università Cattolica del Sacro Cuore, 00168 Rome, Italy

**Keywords:** Signal processing, Biochemistry, Biological techniques, Cell biology, Molecular biology, Physiology, Biomarkers, Diseases

## Abstract

We previously described the profibrogenic effect of NGF on conjunctival Fibroblasts (FBs) and its ability to trigger apoptosis in TGFβ1-induced myofibroblasts (myoFBs). Herein, cell apoptosis/signalling, cytokines’ signature in conditioned media and inflammatory as well as angiogenic pathway were investigated. Experimental myoFBs were exposed to NGF (0.1–100 ng/mL), at defined time-point for confocal and biomolecular analysis. Cells were analysed for apoptotic and cell signalling activation in cell extracts and for some inflammatory and proinflammatory/angiogenic factors’ activations. NGF triggered cJun overexpression and phospho-p65-NFkB nuclear translocation. A decreased Bcl2:Bax ratio and a significant expression of smad7 were confirmed in early AnnexinV-positive myoFBs. A specific protein signature characterised the conditioned media: a dose dependent decrease occurred for IL8, IL6 while a selective increase was observed for VEGF and cyr61 (protein/mRNA). TIMP1 levels were unaffected. Herein, NGF modulation of smad7, the specific IL8 and IL6 as well as VEGF and cyr61 modulation deserve more attention as opening to alternative approaches to counteract fibrosis.

## Introduction

The prolonged survival of myofibroblasts (myoFBs) characterize the pathological process of fibrosis^1,2^. MyoFBs are transient cells arising from quiescent fibroblasts (FBs); at injured zone FBs are exposed to proinflammatory cytokines, growth factors and extracellular matrix (ECM) products/mediators^2^. A defined deletion of myoFBs is vital to ensure a proper tissue healing (physiological remodeling) and the restoration of local function, while the aberrant apoptosis can account for excessive scarring and overt fibrosis^2,3^. Thus the mechanisms of myoFB persistence still require attention.

Nerve Growth Factor (NGF) promotes wound-healing and regulates ECM remodeling, allowing a well-balanced tissue repair and recovery of functional activity^4,5^. This pleiotropic factor orchestrates crucial cell activities (proliferation, stimulation, differentiation and survival) on structural and functional cells belonging to the endocrine, immune and visual systems^4^. NGF binds to both specific (trkA^NGFR^) and/or pan-neurotrophin (p75^NTR^) receptors to promote (autocrine/paracrine) downstream effects on the surrounding epithelial, endothelial, stromal and immune (mast cells, eosinophils, B/T cells, macrophages) cells^6–10^. Previous studies from our laboratory described the profibrogenic effect played by NGF on healthy-control primary cultures of conjunctival Fibroblasts (FBs), mainly expressing trkA^NGFR^, highlighting NGF ability to trigger apoptosis in the in vitro TGFβ1-induced myofibroblast model expressing both trkA^NGFR^/p75^NTR^^11^.

In visual system, the topical (eye-drop) NGF application improved corneal sensitivity and promoted corneal epithelial healing in both moderate and severe neurotrophic keratitis as well as in neurotrophic or autoimmune corneal ulcers^10,12^. A crosstalk between epithelial cell and FB/myoFB cell balance accounts for the correct tissue remodeling, sustained by the immune related cell pattern^6^. Direct paracrine signaling from early and late apoptotic cells in concert with immune cells (macrophages, neutrophils, infiltrating eosinophils and tissue resident mast cells) can also drive fibrotic outcomes, by a specific release of factors inside fibrotic tissue^6^. Therefore, alternative approaches, other than steroid and proper antifibrotic drugs, have been suggested for counteracting overt fibrotic response and promoting a “remodeling again” inside fibrotic tissues^13^. Few reports highlighted the possibility that angiogenesis or vascular regression might counteract the overt fibrosis: early vascular outgrowth or vascular apoptotic cell presence might represent regulators of fibrosis^14^. NGF was found to increase VEGF expression in different cell types including FBs/myoFBs, and in turn VEGF-D was found to stimulate myofibroblast growth, migration and collagen synthesis, opening to other routes to regulate tissue remodeling and fibrosis^14–16^. The cellular matrix protein cyr61 (CCN1), a multifunctional matricellular protein dynamically involved in ECM remodeling, enhances TGFβ1/SMAD profibrotic signaling in fibroblasts and contributes to lung fibrosis and angiogenesis^17^.

Since FBs represent the main target/effector cells of tissue remodeling and NGF is known to sustain the in vitro myoFBs development and modulate apoptosis, further NGF effects model were investigated on this myoFB, in terms of cell signal transduction (cJun, p65-NFkB and smad7), cytokines’ signature in conditioned media and remodeling/angiogenic pathways.

## Materials and methods

### Routine reagents

Plasticware and analytic grade reagents were purchased from Starlab (Milan, Italy), Euroclone (Milan, Italy), ICN Biochemicals (Costa Mesa, CA), Sigma-Aldrich (St. Louis, CA, USA) and Carlo Erba (Milan, Italy), otherwise specified in the text. Sterile tissue culture plasticware was obtained from NUNC (Roskilde, Denmark) and medium were from Euroclone (Milan, Italy). RNAfree MilliQ water (DirectQ; MerckMillipore, Vimdrone, Milan) and home-made buffers (Phosphate-Buffered Saline, PBS: 10 mM phosphate pH 7.5 in 0.9% saline; Tris-Buffered Saline, TBS: 50 mM Trizma pH 7.5 and 160 mM NaCl) for biomolecular analysis were daily autoclaved.

### Conventional and p75^NTR^ siRNA cultures: NGF treatments

The study followed the guidelines of the Declaration of Helsinki and complied with the ARVO Statement in Ophthalmic and Vision Research for research. The study was approved by the intramural scientific committee of IRCCS-Fondazione Bietti. Human conjunctival FBs provided by Innoprot (n = 3; Bizkaia, Spain) were expanded in complete DMEM (10% Fetal Bovine Serum (FBS), 1 mM Glutamine and 1% pen/strep mix)^8,9^. Confluent 48 h-serum starved monolayers were exposed to 10 ng/mL TGFβ1 (PeproTech EC, Ltd., London, UK) over 3 days, to develop “in vitro induced” myofibroblast (myoFBs). Induced myoFBs were replaced at high density in 24-well culture plates (on coverslips) for microscopy as well as in 24-well or 6-well plates for biomolecular analysis. Cells were exposed to increasing (1–100 ng/mL; β-NGF Grade-I; Alomone Labs Ltd, Jerusalem, Israel) NGF concentrations in 0.1% FBS-DMEM, over 15 min–30 min–3 h–6 h–24 h for respectively molecular and biomolecular analysis. After stimulation, cells were washed with Hank’s Balanced Sodium Solution (HBSS, Euroclone) and harvested for biomolecular analysis or quickly fixed for microscopical evaluations. Untreated cells (0 ng/mL) were provided for each experiment route, to minimize biochemical changes alongside subculturing.

Myofibroblasts (30–50% confluence) were transfected with 200 nM antisense p75^NTR^ oligonucleotides (for sequence details see Table 1 Ref.^7^) in lipofectamine RNAiMAX (ThermoFischer, Waltham, MA USA), according to a standard protocol with minor revisions^11^. After 3 days, different doses of NGF were added to both siRNA and sham cultures to estimate growth and morphology after 24 h. The neutralization has been confirmed by immunofluorescence of p75^NTR^ protein expression.

**Table 1:** Antibodies and primers overview.

Target (Human)	Dilution	Host	Specificity (marker)	Source
**A: Biochemical analysis**				
p75^NTR^	1:100	Goat	Pan-neurotrophin p75^NTR^	Santa cruz
αSMA	1:500	Mouse	Smooth muscle actin alpha	Sigma
p65NFkB	1:100	Goat	Nuclear translocation factor	Santa cruz
phospho p65NFkB	1:50	Goat	Ser63-Nuclear translocation factor	Cell Signaling Technology, Leiden, Netherlands
Annexin V	1:100	Mouse	Early apoptotic	Biolegend
cJun	1:1000	Mouse	c-Jun	Cell Signaling
Actin	1:1000	Mouse	Cytoskeleton	Sigma
Tubulin	1:1000	Mouse	Cytoskeleton	Santa Cruz

Target (Human)	Accession	Sequence (left primer)	Tm/amplicon	
**B: Molecular analysis**				
Bcl2	BC027258	ctcccaatactggctctgtc	56 °C/111 bps	
Bax	BM673184	tgccagcaaactggtgct	56 °C/129 bps	
Smad7	AF015261	ccaactgcagactgtccaga	60 °C/107 bps	
Cyr61	BC009199	cacccttctccacttgacc	58 °C/163 bps	
VEGF-A	AF022375	tgacagggaagaggaggaga	59 °C/141 bps	
GAPDH	BC013310	gaaggggtcattgatggcaac	63 °C/100 bps	
H3	AC239868	gtctgcaggctggcatagaag	61 °C/100 bps	

(A) primary antibodies used for western and immunofluorescence assay. (B) Summary of name, for/rev sequence (5′ to 3′), PCR product size (amplicons in bps), annealing conditions and GenBank Accession Numbers of each gene investigated. Amplification profile: hot start activation (95 °C/15 min); 39 cycles: den. at 94 °C/10 s—ann. At 58 °C/15 s—ext. at 72 °C/10 s; melting curve recording 55 °C to 95 °C with one fluorescence reading every 0.5 °C; further ext. 75 °C/5 min.

### Double immunofluorescent analysis

Attached cells (monolayers on coverslips) were washed with HBSS, post-fixed with buffered-PFA (2% paraformaldehyde in autoclaved PBS, quenched with 50 mM NH_4_Cl and briefly blocked/permeabilized in 0.8% BSA and 0.3% Triton X100 in PBS. Incubations with αSMA (1:100; clone AC-15); AnnexinV^FITC^ (1:100; Biolegend, San Diego, CA); goat anti-human p65 subunit of NF-kB (referred as p65NFkB; sc-10741); goat anti-human phospho-Ser63–p65NFkB (referred as phospho-p65NFkB; #9261) and goat anti-human p75^NTR^ (1 µg/mL; sc-6188), as listed in Table 1A, were followed specie-specific fluorophores-conjugated secondary antibodies (1:300/1:500) including Cy2 (green) or Cy5 (blue). Irrelevant isotype-matched IgG antibodies or omission of the first antibodies (Vector Laboratories, Inc., Burlingame, CA) were used as controls for the channel series or background subtraction. Coverslips were placed upside-down on glasses by antifading mounting medium (Vectamount, Vector) for analysis. Single acquisitions and merged staining’s were performed according to fluorescent emission wavelength.

### Confocal image capturing and IntDen analysis

Dry and oil immersion images were acquired by C1 software connected to TE-2000U inverted confocal microscope equipped with lasers or with microscope equipped with epifluorescence and software for image acquisition (Nikon, Tokyo, Japan). For image capturing, 40 × and 60 ×/oil immersion objectives were used, and images were saved/converted into 8-bit TIFF format and subjected to Integrated densitometric (IntDen) analysis (Image J v1.43; NIH -http://rsb.info.nih.gov/ij/), according to a standard procedure^18^. Fluorescence Intensity (FI) was determined by measuring the average fluorescent signal per pixel within a fixed area (round frame) placed over the relevant area of the cell. Averages (means ± SD) were calculated for each data set, using GraphPad Software (Prism 8, San Diego, CA). Representative images were assembled in figures by using the Adobe Photoshop 2020 22.0.0 software release (Adobe Systems Inc., San Jose, CA). Minimal adjustments were made to images to obtain the best quality of presentation.

### Western blotting analysis

Monolayers (10^6^ single cells per well) were prewashed in ice-cold HBSS, cells were detached with a trypsin solution and harvesting was performed in 10% FBS—HBSS to neutralize enzyme activity. Pellets (3500 rpm/3 min) were treated with 70 µL lysis buffer (20 mM Tris, pH 7.5, 150 mM NaCl, 1 mM EDTA, 1% Triton X-100, 10% glycerol, 1 mM NaF, 1 mM Na_3_VO_4_ and a Protease Inhibitor Cocktail (Pierce Biotechnology, Rockford, IL). Cell protein extracts (4 °C for 20 min) were centrifuged (15,000 rpm for 10 min; 4 °C) to discard debris and cytosolic/nuclear protein extracts were simultaneously produced by using the NE-PER Nuclear and Cytoplasmic Extraction Reagents kit (78,833; Pierce), following the protocol recommended by the manufacturer.

Cytosolic and nuclear sub-fractions (3 μL from each sample) were subjected to A280 quantification against lysozyme referring protein (NanoDrop 1000 Spectrophotometer, Thermo Fisher Scientific, MA, USA). Protein extracts were stored at − 20 °C until SDS-PAGE separation and immunoblotting analysis. Appropriate normalized aliquots were prepared for each sample to assure only one freeze–thaw cycle.

Normalized extracts (15–30 µg protein) were diluted in Laemmli sample buffer (62.5 mM Tris, pH 6.8, 25% glycerol, 2% SDS, 0.01% bromophenol blue and 5% ß-mercaptoethanol), heated to 95 °C for 5 min, loaded on precasted 4%/7.5% or 10% gradient polyacrylamide minigels and run at 150 mV to frontline in electrophoresis buffer (all from Bio-Rad, Hercules, CA). Bands were transferred (15 mV/15 min for 2 membranes) onto nitrocellulose membranes (protein high affinity; GE-healthcare, Chicago, USA) in appropriate transfer buffer (48 mM Tris pH 6.8, 39 mM glycine, 0.00375% SDS and 20% methanol) by using the semidry transblotting procedure (Invitrogen, California, USA). For immunoblotting, membranes were washed in tris-buffered saline (TBS: 50 mM TrisHCl and 0.9% NaCl; pH 7.5), blocked in TBS containing 5% non-fat dry milk and 0.03% Tween-20 (benchtop/60 min) and probed (18 h/4 °C) with anti-human c-Jun (1:1000; #9162); p65NFkB (1:1000) and phospho-p65NFkB (1:700) all listed in Table 1A. After washing in TBS, membranes were incubated with horseradish peroxidase-conjugated goat anti specie-specific IgG antibodies (1:20,000; Jackson ImmunoResearch) under gentle rotation (60 min/300 rpm). The immunolabeled bands were visualized after incubation with enhanced chemiluminescent ECL Pico Western Bot solution (Pierce) and digital capturing by the 1D Kodak Image Analysis Software (Kodak 550 Imager station; Eastman Kodak Company, Sci. Imaging Systems, Rochester, NY). After acquisitions, membranes were treated at 56 °C for 60 min in stripping solution (62.5 mM Tris pH 6.7 containing 2% SDS and 100 mM 2-mercaptoethanol) and re-probed with actin or tubulin to confirm equal protein loading. Digital images were subjected to densitometric analysis and final images were assembled with Adobe photoshop 2020.

### Ella microfluidics-based platform

Specific expression of Cyr61, IL6, IL8, TIMP1 and MMP9 was assessed by using the multiplex Ella platform. Briefly, 1:2 diluted samples were loaded on to the cartridge, according to a standard procedure provided by the manufacturers (Protein Simple, CA, USA). All steps in the procedure were run automatically by the instrument with no user activity. Cartridges include a built-in lot-specific standard curve for the defined protein. The obtained data were displayed as pg/mL and automatically calculated by the internal instrument software.

### Chip-based protein microarrays

Conditioned media from myoFBs were hybridized in chip arrays and labeled according to the manufacturer’s instructions (G-series arrays; Ray Biotech, Norcross, CA). Each cy3-labeled treated/untreated sample was combined with an equal amount of pooled cy5-labeled common reference and 70 µL mixture (well/chip) was hybridized for 18 h at 4 °C. After washes under stringency, the glass slides were washed once in MilliQ water to remove salts and quickly spun to dry the chips. Slides were analyzed in parallel: double fluorescence signals were acquired with the GenePix 4100 microarray scanner (Molecular Devices LLC, Sunnyvale, CA) equipped with the GenePix Pro 3.0 software (Axon Instruments, Foster City, CA). Data were expressed as ratio (treated/untreated signal).

### RNA extraction, cDNA synthesis and relative real-time PCR

Total RNA was extracted from 6-well plates cells according to the TRIfast technique (Euroclone), treated with DNAseI (AB1709; Ambion Inc., Austin, TX), spectrophotometrically analyzed for quantification and purity (260/280 ratio; Nanodrop A1000 Spectrophotometer; Celbio, Milan, Italy). A further RNA quality assessment was carried out on randomly selected RNA extracts in a 1% agarose (Promega, Milan, Italy) gel supplemented with Ethidium Bromide (ICN). Only samples with 260/280 > 1.8 were used for amplification studies.

cDNA was generated from 3 µg total RNAs by using the GoScript standardized procedure (Promega) in the presence of random hexamers (Promega), in a OneCycler programmable thermocycler (Peqlab; VWR Radnor, Pennsylvania, USA). For amplifications, 3 µL cDNAs for target gene and 1µL cDNAs for referring ones were amplified in a 20 µL final volume of SYBR Green PCR mixture (Applied Biosystems, Foster City, CA), using the Opticon2 real time thermocycler (MJ Research, Watertown, MA). Amplification profile was: one cycle of 95 °C/15 min initial denaturation followed by 35–45 cycles at 95 °C/30 s (denaturation), 55–60 °C/25 s (annealing) and 72 °C/30 s (elongation), followed by fluorescence monitoring at 60–90 °C, 0.01 °C for 0.3 s and further elongation at 72 °C/5 min. Negative and positive controls were run in parallel, according to a standard procedure. The specific primer pairs, the accession number and the length of amplicons are shown in Table 1B (synthesized by Eurofin MWG, Ebersberg, Germany).

Only normalized samples were amplified and cycle threshold (Ct) values from good melting curves were used for analysis in the REST software^19^. Relative gene expression was calculated as the expression level of target gene with respect to that of referring genes (H3 and/or GAPDH), considering both treated *vs.* untreated cells. As fold-change ratios were expressed in 2-log, only increase/decrease over 2 were considered of interest in the statistical evaluation.

### Statistical analysis

Experiments were performed in triplicate, starting from n = 3 primary sets expanded and used in the 5th–7th generation range, and analysed for three times to validate results. Controls (without NGF stimulation) were carried out in every plate and each generation. Descriptive statistic and graphics were performed using the GraphPad Prism 8.01 (GraphPad, San Diego, CA). The standard deviation (SD) and Standard Error from the Mean (SEM) were calculated to assess the variations between different treatments under the same conditions (untreated cells). Data were subject to statistical significance by one-way analysis of variance (ANOVA) using the StatView II Software (Barckley, CA). ANOVA was coupled to post-hoc analysis performed using Tukey's test between subgroups and with respect to untreated cells). All p values ≤ 0.05 were considered as significant and depending on post-hoc analysis, appropriate asterisks were used in panels as follows: *p < .05; **p < .01 and ***p < .001 (highly significant). For chip array analysis, a *p* < .0083 was used together with a limit of 2-folds. Data are mean ± SD (text) and mean ± SEM (figures), and error bars were calculated from at least three independent experimental sets.

## Results

**Figure 1:**
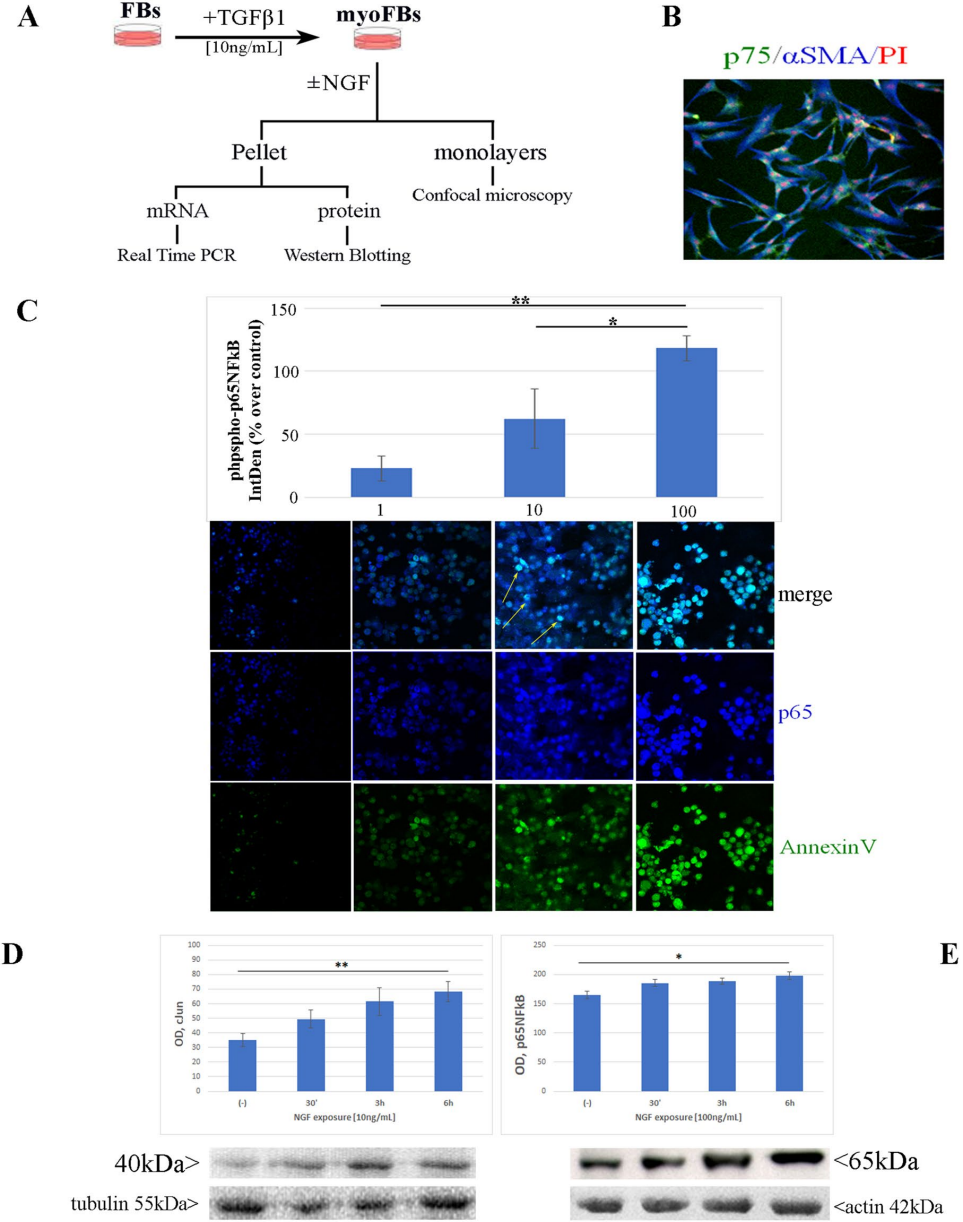
Overall experiment and cell features. (**A**) Flow chart summarizing the TGFβ1-induced myoFB and NGF exposures/analysis. Stimuli and timing are highlighted. (**B**) Representative confocal microscopy image showing co-expression of p75^NTR^ (cy2/green) and αSMA (cy5/blue) on nuclear stained myoFBs (Propidium Iodide/red) (magnification, × 200). (**C**) NGF triggers p65NFkB expression in AnnexinV positive myoFBs. Dose-dependent increase of total p65NFkB is shown over 24 h, as detected by quantitative Imaging analysis (IntDen) on single-channel p65NFkB (cy2/green) confocal images. Immunoreactivity for AnnexinV (cy2/green) and p65NFkB (cy5/blue) in myoFBs as shown in the overlay row and the single panels below reported, all with respect to NGF exposure concentrations. Arrows in 10 ng/mL NGF (merge) panel point at some coexpressing cells: NGF effect is detectable as soon as at 10 ng/mL and significantly increase at 100 ng/mL doses. (DE) Time-dependent histograms and cropped blots, showing expression of cJun and p65NFkB,at the specific molecular weight for cJun (40 kDa; **D**) and total p65NFkB (65 kDa; **E**) above the related tubulin (55 kDa) and actin (42 kDa) housekeeping ones, from NGF exposed myoFBs over a timing of 30 min, 3 and 6 h. Densitometric analysis was performed specifically on the cropped image (see panel), resulting from the original immunoblot (see Fig. S1), to avoid any artefact in acquisition and data were expressed as compared to untreated ones. Minor brightness/contrast adjustment were performed by photoshop for better image presentation (cropped panel). ANOVA analysis followed by Tukey Kramer post-hoc; Sign: *p < .05; magnification, × 400.

A flow chart of overall experimental procedure is shown in Fig. 1A. High density replated and serum-starved confluent TGFβ1-induced myoFBs (herein shorten as myoFBs) were exposed to single or chronic increasing NGF concentration (0–100 ng/mL), over 15 min–30 min–3 h–6 h–24 h (protein/mRNA) time-point sets. Untreated myoFBs were used as control and carried out at each set of experiments. The typical spindle-appearance and the p75^NTR^ (green) and αSMA (blue) expression on red nuclear counterstaining are visible in Fig. 1B.

### NGF activates p65NFkB / cJun molecules in AnnexinV positive myoFBs

NGF induced a dose-dependent increase of phospho-p65NFkB protein in AnnexinV positive myoFBs. The quantification of immunoreactivity (Integrated Density; IntDen) is shown in the histogram (Fig. 1C). IntDen values are % over control (sister untreated myoFBs). Representative confocal images are shown in Fig. 1C reporting both single (specific) and double (merge) immunostainings. The merge visualization in the upper row (Fig. 1C) highlight that NGF exposed myoFBs positively stained for phospho-p65NFkB (blue/cy5) and AnnexinV (green/FITC). Single immunoreactions for phospho-p65NFkB (middle row, blue/cy5) and AnnexinV (lower row; green/FITC) are shown below. This p65NFkB expression was associated with a significant increase of enlarged, nuclear condensed cells, especially after repeated NGF treatment (see white arrows). TGFβ1 by itself did not trigger significant p75^NTR^ expression nor AnnexinV, retaining a high trkA^NGFR^/p75^NTR^ ratio (data not shown). At the same time, NGF exposure induced the activation of cJun and NFkB pathways in a time-dependent fashion. Data from western blotting analysis corroborated the confocal ones, at least at 100 ng/mL NGF exposure. As shown in Fig. 1D, NGF triggered the cJun protein expression in a time-dependent fashion, as compared to untreated ones (**p < .001 vs. untreated cell extracts; ANOVA-Tukey Kramer post-hoc). In line, p65NFkB protein signal also increased in a time-dependent fashion (*p < .05 vs untreated cell extracts; ANOVA-Tukey Kramer post-hoc; Fig. 1E). Original immunoblots for cJun and p65NFkB are visible in the supplementary Fig. S1.

### NGF promotes nuclear translocation of phosphorylated p65NFkB in receptive myoFBs

**Figure 2:**
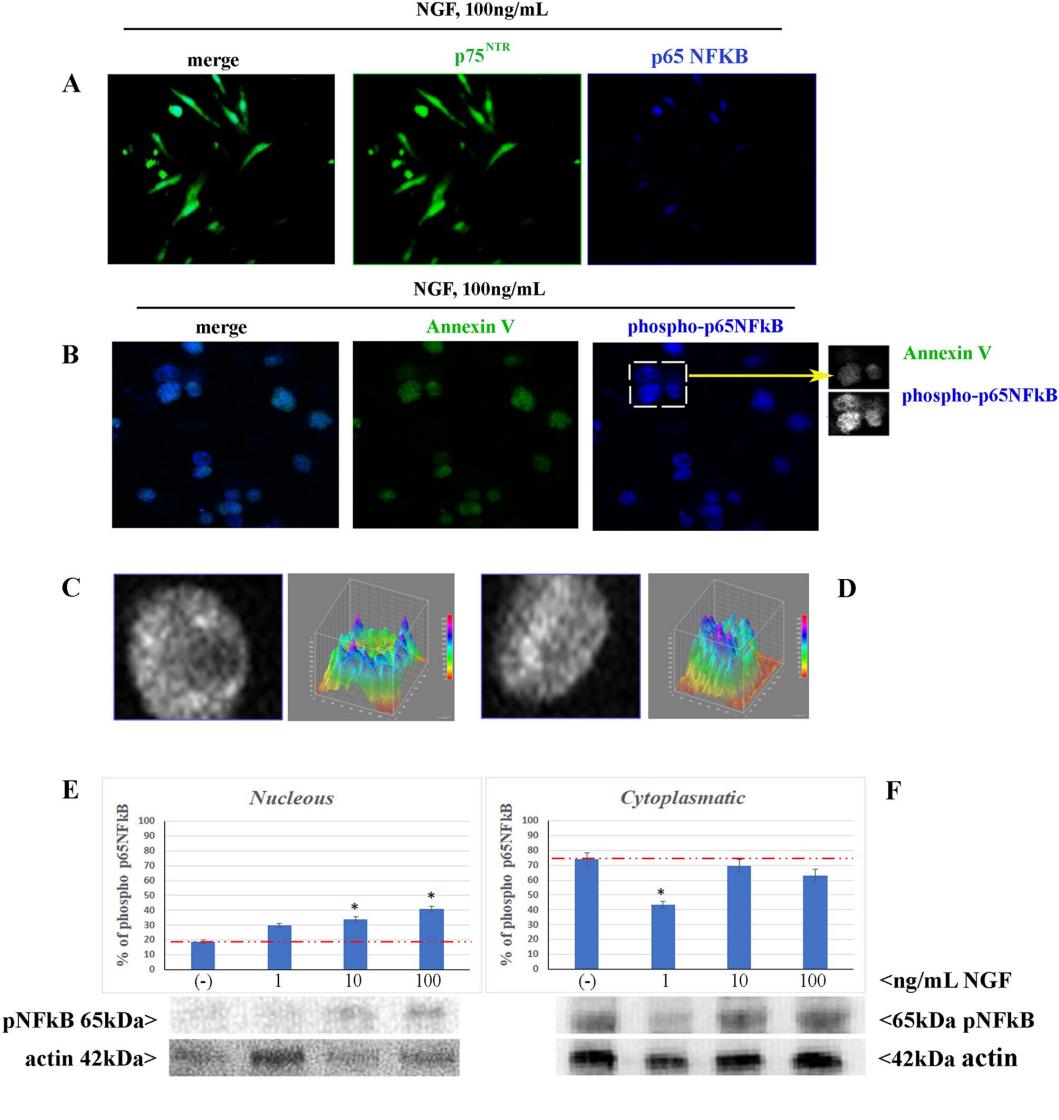
NGF triggers p65-NFkB phosphorylation and nuclear translocation. Cells were exposed to repeated (chronic) 100 ng/mL stimulation and sampled at 24 h for phosphorylation and translocation analysis of p65NFkB. (**A**) Representative confocal image showing the co-expression of p75^NTR^ and p65NFkB in induced myoFBs (yellow; overlay/left panel). (**B**) Representative higher magnification overlay images (left panel) displaying AnnexinV-positive myoFBs (cy2/green; labelling the outset leaflet of the plasma membrane) with nuclear localization of phospho-p65NFkB (cy5/blue). Single acquisitions are shown in middle (AnnexinV; cy2/green) and right (phospho-p65NFkB; cy5blue) panels. Gray level images from a selected cell in right (**B**) panel, as pointed by arrow. (**C**,**D**) Representative expression of different intracellular localization expressed as pseudocolor carried out by single cell densitometric analysis. As shown by pseudocolor histograms, the nuclear phospho-p65NFkB expression was more evident in AnnexinV positive cells (green; **D** vs. **C**), indicating a selective activation/translocation. (**E**,**F**) Quantification of nuclear vs. cytoplasmatic phospho-p65NFkB expression in protein extract fractions upon increasing NGF exposure (24 h). Red dash lines indicate baseline expression (untreated cells). The densitometric analysis was performed specifically on the cropped image (see panel), resulting from the original immunoblot (see Fig. S1), to avoid any artefact in acquisition and data were expressed as compared to untreated ones. Minor brightness/contrast adjustment were performed by photoshop for better image presentation (see cropped panels probed with p65NFkB or actin). Data are mean ± SEM of three independent experiments. ANOVA analysis followed by Tukey–Kramer post-hoc; sign: *p < .05. Magnifications: a–b, × 400; c, × 600.

Immunoreactivity for p65NFkB and p75^NTR^ (merge, left panel) was observed in myoFBs exposed to repeated 100 ng/mL NGF stimulation (Fig. 2A). Single staining for p75^NTR^ (middle panel/green/cy2) and p65NFkB (right panel/blue/cy5) are also shown. MyoFBs showed a significant increase in phospho-p65NFkB expression at both cytoplasm and nuclear levels in AnnexinV positive (green/FITC) cells (Fig. 2B). The different AnnexinV (upper frame) and phospho-p65NFkB (lower frame) expression is shown (grayscale of the two cells framed in overlay image is shown in the right side of Fig. 2B). Single cell densitometric analysis was carried out specifically for phospho-p65NFkB in AnnexinV negative and positive cells. A representative expression of different cytoplasm to intracellular localization of phospho-p65NFkB is reported, as shown by greyscale acquired and pseudocolors cell images (Fig. 2C,D). As shown by 3D IntDen graphical representation (right) panels, the intranuclear phospho-p65NFkB expression was more evident in AnnexinV positive cells (Fig. 2D) with respect to AnnexinV negative one (Fig. 2C).

Western Blotting analysis confirmed the increased expression and nuclear translocation of phospho-p65NFkB protein, as shown by graphics and a representative immunoblot on both nuclear (Fig. 2E) and cytoplasmic (Fig. 2F) extracts. Quantifications were carried out against untreated nuclear and cytoplasm extracts showing a significant increase in phospho-p65NFkB in nuclear extracts in a dose dependent fashion, as visualized over a baseline dashed line in the bar-graphs (*p < .05 in 100 ng/mL exposed cells with respect to untreated ones; Fig. 2E). A decrease in cytoplasm expression was monitored, with the lowest expression at 1 ng/mL NGF, without explanation (Fig. 2F).

**Figure 3:**
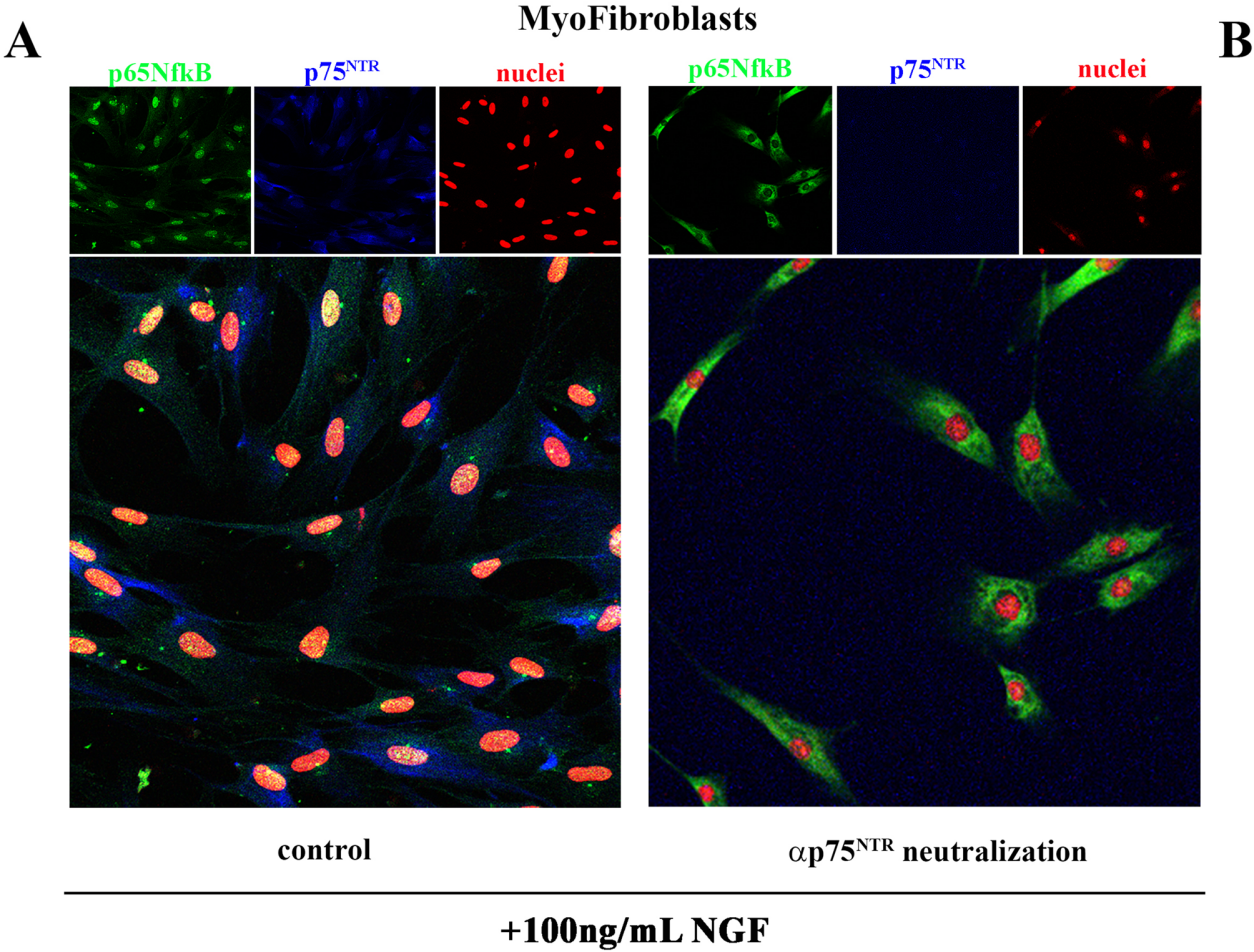
NGF driven p65NFkB expression/localization. Confocal images of p75^NTR^siRNA cultures were acquired at 24 h from NGF stimulation. Representative images of p65NFkB (green) and p75^NTR^ (blue) immunoreactivity over red nuclei (propidium iodide) of 100 ng/mL NGF-exposed myofibroblasts, either untreated (**A**) and p75^NTR^ oligonucleotides transfected ones (**B**). Upper squares display single staining. Images are representative of three independent experiments acquired under the same channel-series parameters by confocal microscope Nikon c*i* and analyzed by NIS software.

Confocal microscopy showed the localization of perinuclear p75^NTR^ and p65NFkB immunoreactivity inside nuclei after NGF treatment (100 ng/mL; Fig. 3A). By contrary, p75^NTR^ siRNA (oligonucleotides) transfected myofibroblasts showed a drastic reduction of p75^NTR^ immunoreactivity, which resulted in a retained cytoplasm immunoreactivity of p65NFkB (Fig. 3B). The original immunoblots specific for both cytoplasmatic and nuclear expression phospho p65NFkB are shown in Fig. S1.

### NGF modulates Bcl2:Bax ratio and Smad7 transcripts

**Figure 4:**
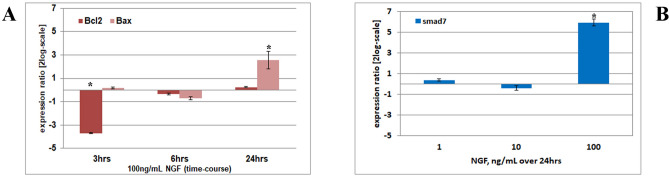
NGF and Bcl2/Bax associated apoptosis. NGF modulates Bcl2/Bax transcript ratio (**A**; time response) and Smad7 (**B**; dose response) transcript expression. Real time PCR followed by REST-ANOVA coupled analysis are shown for 100 ng/mL NGF treated cells, as compared to untreated ones*.* Both significant NGF modulation of Bax transcript and smad7 transcript expression are visible (*). Note that smad7 works as inhibitory factor of TGFβ1 signaling. Data are mean ± SEM (fold changes as log2-expression) of three independent experiments.

To explore the possible role of Bcl2 family members in the NGF-induced apoptosis, the effects of NGF on the expression of Bcl2 and Bax protein was examined by real time PCR. Bcl2 and Bax proteins are well-known anti-apoptotic and pro-apoptotic in situ markers, with counteracting effects (Bcl2 acts by blocking Bax-induced apoptosis)^20^. As shown in Fig. 4A, changes in expression of Bcl2 and Bax transcripts were monitored upon 100 ng/mL NGF exposure. Particularly, Bcl2mRNA deregulation was higher at 3 h (− 3.691 ± 0.037_log2_) while Bax transcript upregulation (2.547 ± 0.749_log2_, *p < .05) was maximum at 24 h, as observed by REST-ANOVA coupled analysis on treated versus untreated RNA extracts (Fig. 4A). As shown in Fig. 4B, smad7 transcript (the inhibitory factor of TGFβ1 signaling) was significantly increased at 24 h upon 100 ng/mL NGF exposure (5.946 ± 3.320_log2_, *p < .05; fold changes vs. untreated expression; REST-ANOVA coupled analysis).

### NGF influences myoFBs’ protein signature (conditioned media)

**Figure 5:**
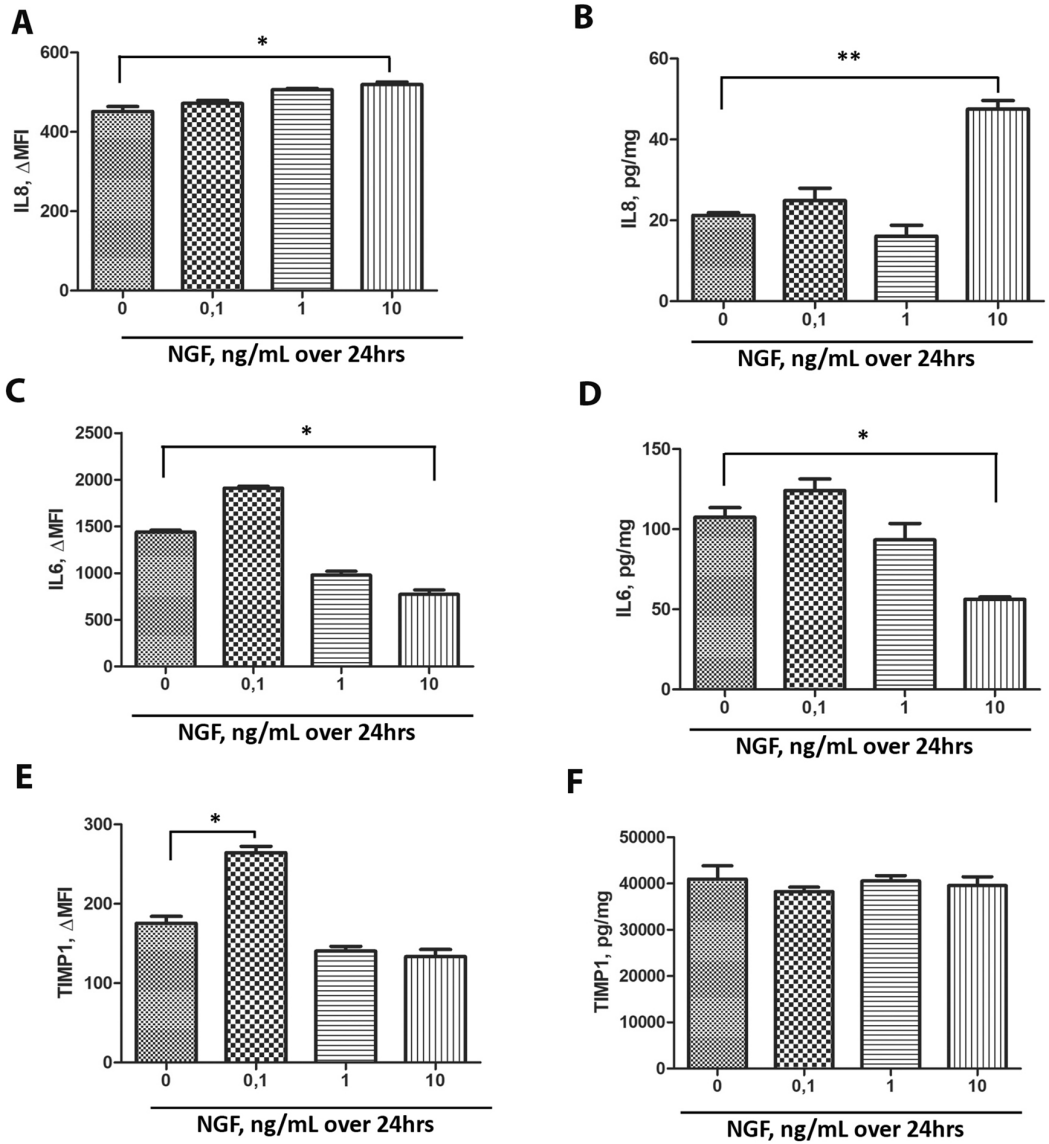
Overview of the in vitro results obtained by microarray chip analysis and ELLA microfluidic assay. Bar-graph showing the expression of IL8 (**A**,**B**), IL6 (**C**,**D**) and TIMP1 (**E**,**F**) in conditioned media from myoFBs exposed to increasing NGF doses (0–10 ng/mL). Note the increase of IL8, the decrease of IL6 and the quite unchanged expression of TIMP1. Data are mean ± SEM (fold changes or pg/mL) of three independent experiments. ANOVA analysis followed by Tukey–Kramer post-hoc; sign: *p < .05 and **/ < .01, as for multiparametric array analysis.

To identify potential mediator between inflammatory (cytokines, chemokines, adhesion molecules), growth (neurotrophins, fibrogenic and angiogenic factors) and tissue remodeling (TIMPs) ones, potentially produced/released into conditioned media by NGF-exposed myoFBs, a chip array with sixty (60) potential candidates was personalized. According to multiple comparison and Bonferroni’s corrections, ANOVA analysis pointed to 3 out of 60 pre-selected candidates. A trend to an increase and significant expression at 10 ng/mL NGF was observed for IL8 (*p* < .001) proteins (Fig. 5A,B). A decrease of IL6 was assessed at increasing NGF doses (*p* < .01; Fig. 5C,D). Besides an increase at lower concentration (Fig. 5E), no significant changes were observed for TIMP1 expression (Fig. 5E,F). Protein chip array data (Fig. 5A,C,E) were confirmed by the ELLA microfluidic platform (Fig. 5B,D,F).

### NGF increased VEGF and cyr61 proteins/gene transcripts

**Figure 6:**
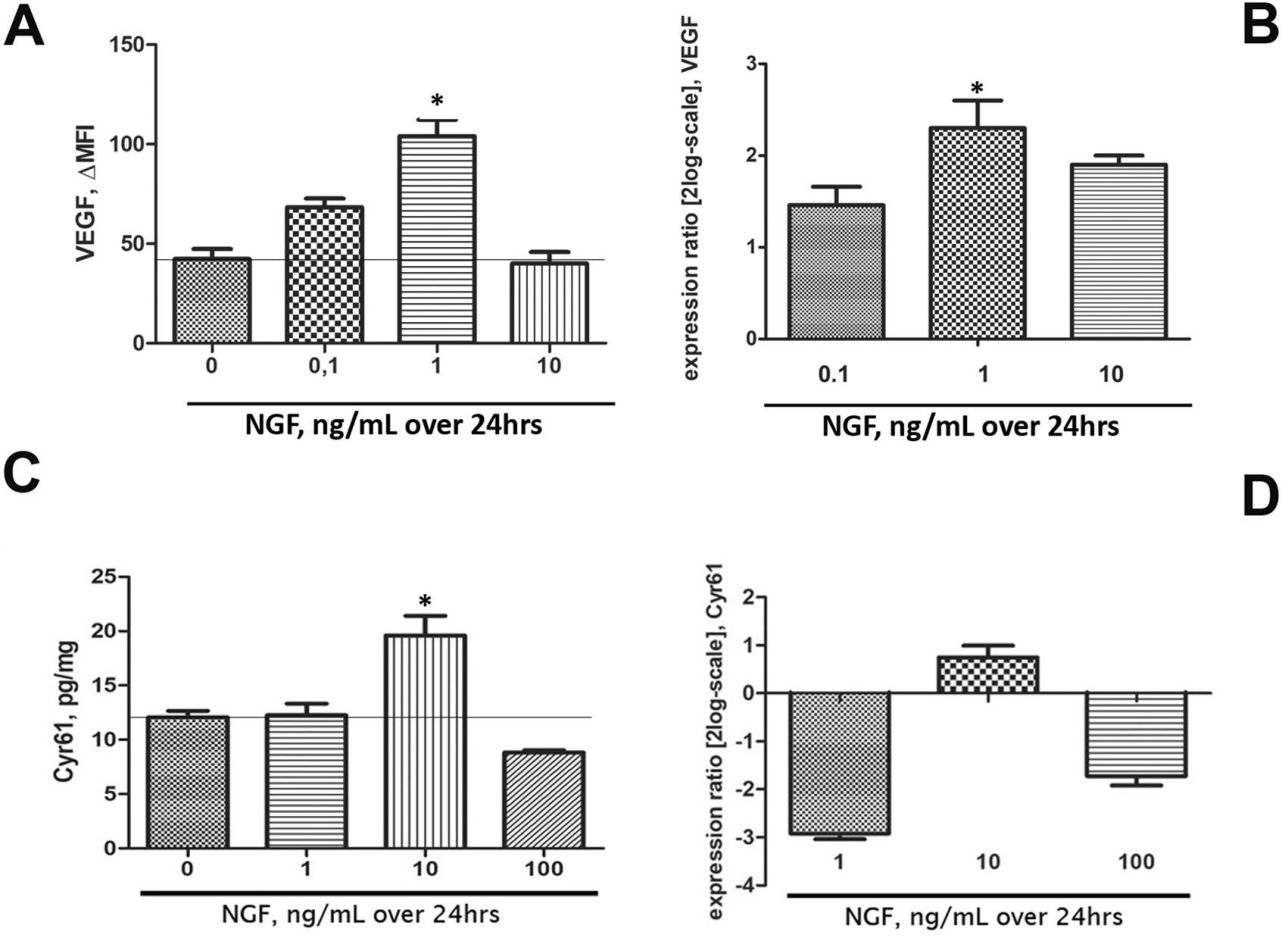
NGF triggers VEGF and cyr61 transcript/protein expression. Protein quantification (**A**,**C**) and transcript amplification (**B**,**D**) specific for VEGF and Cyr61. (**A**,**B**) Bar-graph showing the expression of VEGF: note the higher protein expression after 1 ng/mL NGF exposure, as confirmed at the biomolecular levels. (**C**,**D**) Bar-graph showing the expression of cyr61: note the higher protein expression at 10 ng/mL NGF dose, as confirmed by biomolecular analysis. Data are mean ± SEM (MFI or pg/mL for proteins and fold changes as log2-expression for transcripts) of three independent experiments. ANOVA analysis followed by Tukey–Kramer post-hoc; sign: *p < .05 and **p < .01, as for multiparametric array analysis.

To verify the NGF contribution in angiogenesis, both VEGF and cyr61 molecules were analyzed in conditioned media (protein) and cell extracts (mRNA). Of interest, specific increase of VEGF protein was quantified at 1 ng/mL NGF exposure (Fig. 6A), as confirmed by ELISA (data not shown), and sustained by molecular analysis (Fig. 6B). Of interest, a significant increase of cyr61 protein was detected at 10 ng/mL NGF, as compared to untreated ones (*p* < .05; Fig. 6C) and confirmed by molecular data showing a specific cyr61mRNA upregulation after 1 ng/mL NGF exposure (− 2.918 log; *p* > .05, Fig. 6D).

## Discussion

The NGF healing properties have been prospected since 50s’, from NGF-driven wound healing under physiological states to tissue repair under acute and chronic inflammatory conditions, either Th1 or Th2 driven^6,7^. Herein, our findings extend previous data on NGF-induced apoptosis in trkA^NGFR^/p75^NTR^-bearing and αSMA-expressing myoFBs^11^, by providing evidence on cJun activation, Bcl2:Bad ratio reduction (Bad overexpression), p65NFkB nuclear translocation and smad7 overexpression. The protein signature of conditioned media indicates that long-lasting myoFBs synthesize and release IL8 and VEGF as well as cyr61. Of interest, IL6 and TIMP1 were significantly deregulated.

**Figure 7:**
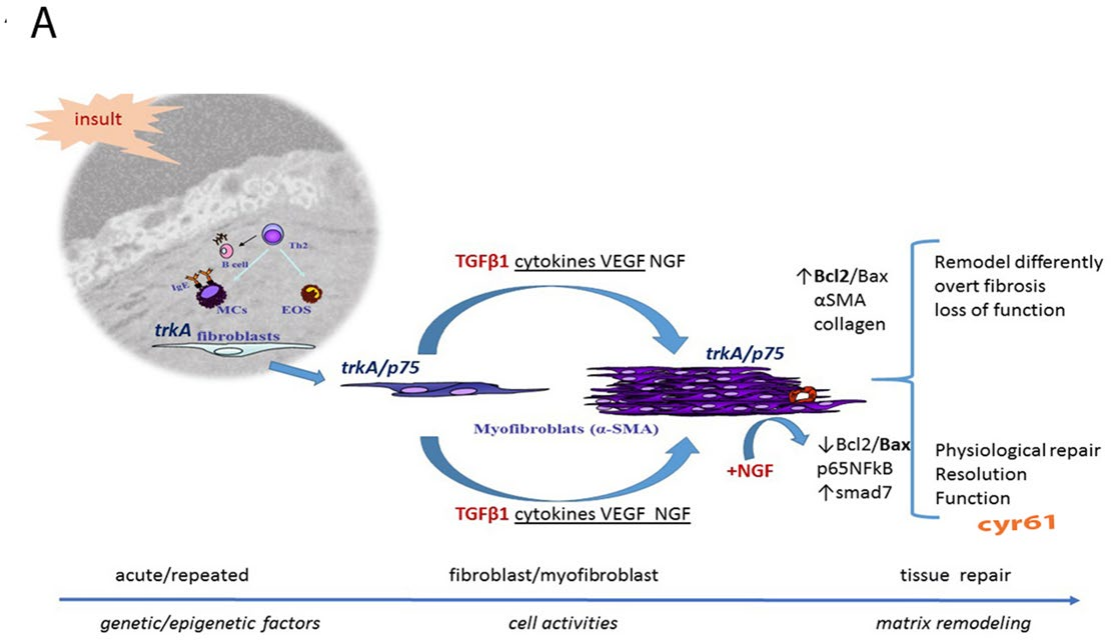
Graphical sketch for a possible explanation for the finding on induced myoFBs upon NGF exposure. In injured tissues, trkA^NGFR^/p75^NTR^- myoFBs arising from resident “quiescent” trkA^NGFR^-FBs (fibroblasts), mainly upon TGFβ1 stimulation and a specific microenvironment, achieve appropriate tissue remodeling (collagen production, ECM refining and contraction). NGF might function as a modulator, in concert with a plethora of inflammatory and matrix remodeling mediators. Overt fibrosis indicates a not well-balanced tissue remodeling. As known, smad7 works as an inhibitory factor in TGFβ1 pathway. Exogenous NGF administration (shorten as + NGF in red) might revert Bcl2/Bax ratio and promotes p65NFkB nuclear translocation restoring physiological condition and function of the tissue, with the resolution of fibrosis. As pleiotropic factor, NGF can also drive neovessel formation and trough VEGF and cyr61 (red) modulation, it might contribute to functional recovery. For representative purpose (on left upper side), a B/W image (a conjunctival frame from a section belonging to a very old historical collection; Anatomia Patologica, Università Cattolica del Sacro Cuore) as background for layered inflammatory cells. Illustration was developed on Microsoft Office Power Point 365 software (Microsoft corp., New York, NY, USA).

A graphical explanation of these findings is shown in Fig. 7. Prolonged stimulation might trigger survival of myoFBs and inappropriate ECM remodeling (overt fibrosis), causing irreversible alterations of organ anatomy and function^21–23^. Apoptotic process represents a physiological strategy removing long-lasting myoFBs, the source of ECM deposition and prolonged matrix contraction^2,3,20,24^. We previously showed the profibrogenic NGF effects on primary cultures of FBs outgrew from skin, lung and conjunctival/corneal tissue (NGF/TGFβ1 expression, αSMA protein metabolism and contractive activity)^11^. Subsequently, we observed that NGF-treated myoFBs showed apoptosis restricted to a p75^NTR^ expressing myoFB phenotype, an effect that was counteracted by specific trkA^NGFR^ and/or p75^NTR^ inhibitors^11,25^. Herein, our findings point at the NGF-mediated cJun increase and Bcl2;Bax ratio decrease in apoptotic myoFBs, supporting previous studies on p75^NTR^-transduced apoptosis in association with Rac-GTPase and c-Jun activation^26^. The observation of a decreased Bcl2:Bax ratio in Annexin positive myoFBs would suggest that Bcl2:Bax ratio might serve as a rheostat to determine the susceptibility to death process^27–29^. Our NGF-mediated apoptotic effect was also associated with p65NFkB nuclear translocation. NFkB transcription factor is composed of p50/p65 subunits close to cytoplasmic IkB inhibitors to prevent nuclear translocation, a route observed in several cell types in the presence of the pan-neurotrophin p75^NTR^ receptor activation^30,31^. Our finding on NGF-mediated p65-NFkB nuclear translocation in these myoFBs strongly suggest the ability of NGF to increase myoFB apoptosis while in unresponsive myoFBs it might modulate the release of some proinflammatory as well as pro-angiogenic factors, through a p75^NTR^ mediated signal^11^. The p75^NTR^ neutralization showed a retaining in p65NFkB translocation, corroborating the NGF/p75^NTR^ specific involvement. We hypothesize that the unresponsive cells were those with a lower trkA^NGFR^/p75^NTR^ ratio (likewise with high Bcl2:Bax ratio).

It is noteworthy highlight that tissues remodeling is strongly driven by TGFβ1 working on a microenvironment enriched of others profibrogenic mediators released by accessory and immune cells, epithelial and endothelial cells and even activated myoFBs^13,17^. The profibrogenic TGFβ1-RI/RII routes the down streaming of smad2/3 and smad4 activation in the presence of a blocked Smad7 activity^32,33^. As observed in this in vitro model, NGF might dampen TGFβ1 signaling thought activation of smad7 expression, explaining at least in part the physiological remodeling observed upon exogenous addition of NGF (corneal ulcers’ closure)^10^. As known, smad7 works as an inhibitory factor in TGFβ1 pathway, the Smad7 expression in these myoFBs might justify at least in part the absence of insistent myoFB signaling in human tissues when repair is NGF assisted^32^. As illustrated in Fig. 7, exogenous NGF administration (shorten as + NGF in red) might revert Bcl2:Bax ratio of fibrotic myoFBs and promote p65NFkB nuclear translocation restoring physiological condition and function of tissue, with resolution of fibrosis.

To identify potential mediator between inflammatory (cytokines, chemokines, adhesion molecules), growth (neurotrophins, fibrogenic and angiogenic factors) and tissue remodeling ones, we used a customized array chip approach with the screening of 60 potential candidates, as previously tested in other studies^34^. Herein, the increased expression of IL8 and the decreased expression of IL6, as detected in conditioned media of NGF-exposed myoFBs, would imply that NGF does not contribute to the profibrogenic microenvironment.

The dual-faced of angiogenesis at induction of fibrogenesis and resolution of fibrosis has been described in the last years^35,36^. Angiogenesis—the formation of new blood vessels from pre-existing vessels—is a complex and dynamic process occurring both physiologically and pathologically^37^. The tissue healing with proper matrix remodelling would benefit from a controlled induction of vascular activity. A controlled apoptosis and a tidy epithelia-stroma interaction should occur at involved and uninvolved surrounding tissues^36,37^. Some recent evidence suggested that experimental inhibition of angiogenesis ameliorates the development of liver fibrosis, while other recent studies indicate that neutralization or genetic ablation of VEGF can delay tissue repair and fibrosis resolution in damaged tissues^38,39^. The imbalance between pro/anti- angiogenic mediators might contribute to apoptotic or sustain the process of fibrosis, as observed for CCN1/CYR61 involved in attenuating and/or scavenging of TGFβ, mitigating the process of fibrogenesis^13,40,41^. The findings herein reported suggest that the NGF mechanisms might involve a deregulation of TGFβ1 signalling, due to Smad7 gene expression, and alternative a VEGF/cyr61 activation^17,32,41^.

Some open questions still persist (1) as in vitro study on TGFβ1-induced myoFBs, and as known TGFβ1 does not represent the lone differentiating factor in vivo and other soluble mediators can trigger this differentiation; (2) the trkA^NGFR^/p75^NTR^ heterodimer distribution on cell membrane, as other trkA^NGFR^/trkA^NGFR^ and p75^NTR^/p75^NTR^ homodimers can also occur influencing the cellular pathway and finally (3) this NGF-driven p65 NFkB translocation could result in three different pathways (the canonical, the non-canonical, and the atypical one) depending on various upstream activating signals (TNFα, Interleukins, LPS/LT, UV and NO), influencing the entity of Bcl2:Bax ratio^42^. On the contrary, the possibility to use topical NGF will reduce all the disappointed effects played by the circulating addition of NGF.

Taken together, several mediators of inflammation and tissue remodelling, either endogenous or exogenously administered (including natural (omega 3/5) and synthetic ones), might participate in the myoFB-driven remodelling differentially over again, representing candidate factors for driving the correct repair^43^. The NGF-driven upregulation of smad7, an inhibitory component of TGFβ1 pathway, and VEGF/cyr61 deserve further investigation, as opening to alternative approaches in counteracting fibrosis, especially for fibrotic eye diseases.

## Supplementary Information

Supplementary Information.

## Data Availability

All data are available in the manuscript.
