# Supplementary figures and images for: Nerve Growth Factor (NGF) modulates in vitro induced myofibroblasts by highlighting a differential protein signature
Source: Sci Rep. 2021 Jan 18;11:1672. doi: 10.1038/s41598-021-81040-x (PMC7814037; doi:10.1038/s41598-021-81040-x)

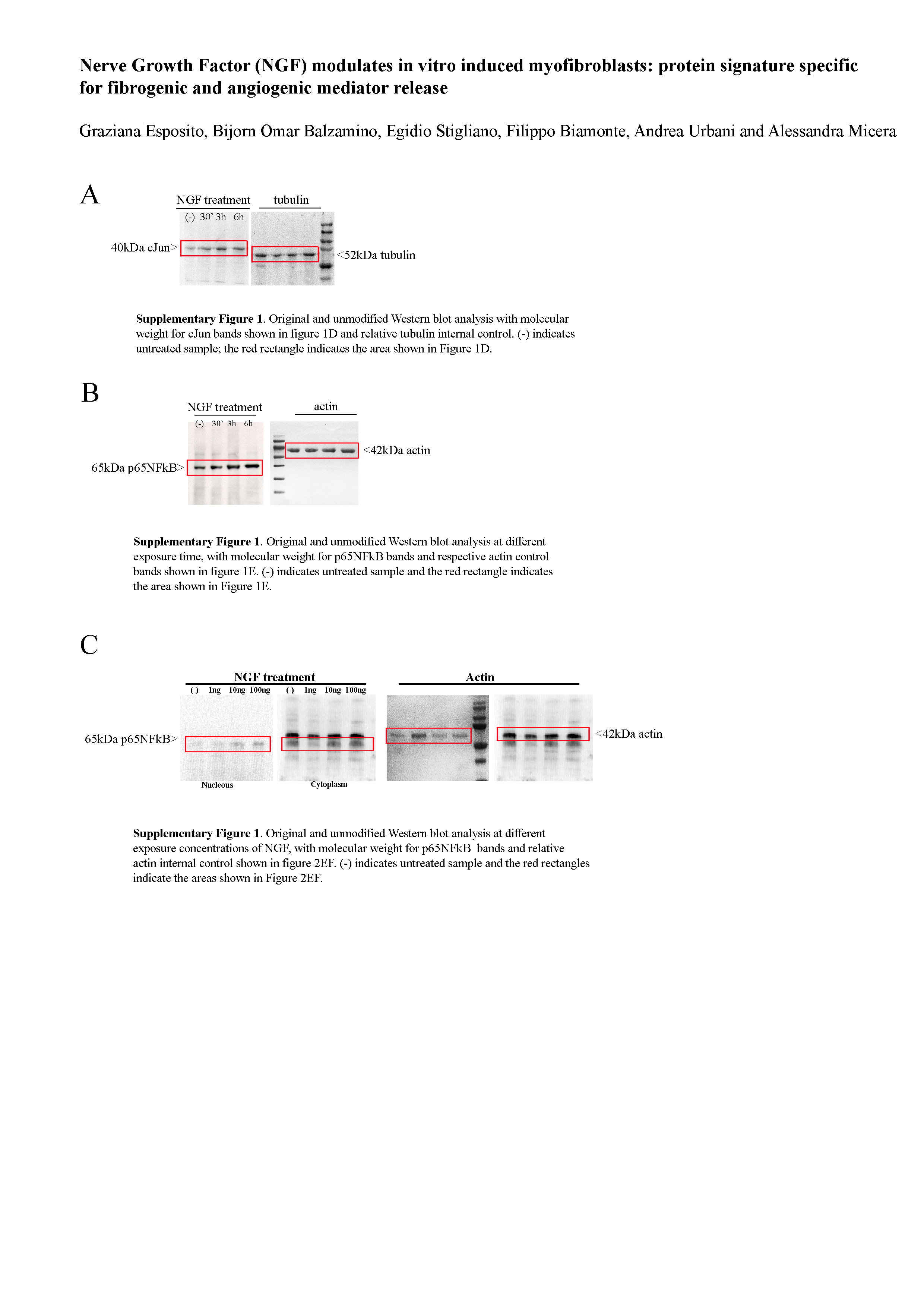

Supplement: Supplementary file 1 — Supplementary Information. [file 41598_2021_81040_MOESM1_ESM.jpg]
